# Correlation between Thyroid Homeostasis and Obesity in Subclinical Hypothyroidism: Community-Based Cross-Sectional Research

**DOI:** 10.1155/2021/6663553

**Published:** 2021-05-29

**Authors:** Yu Zhou, Sujie Ke, Kejun Wu, Jingze Huang, Xuelin Gao, Beibei Li, Xiaoying Lin, Xiaohong Liu, Xiaoying Liu, Li Ma, Linxi Wang, Li Wu, Lijuan Wu, Chengwen Xie, Junjun Xu, Yanping Wang, Libin Liu

**Affiliations:** ^1^Department of Endocrinology and Metabolism, Fujian Medical University Union Hospital, Fuzhou 350001, Fujian, China; ^2^Department of Clinical Pharmacy and Pharmacy Administration, School of Pharmacy, Fujian Medical University, Fuzhou 350122, Fujian, China

## Abstract

**Objective:**

It remains unknown whether obesity has an effect on the pituitary-thyroid feedback control axis in subclinical hypothyroidism (SCH). We aimed to investigate the association of thyroid homeostasis with obesity in a SCH population.

**Methods:**

Our study consisted of a community-based and cross-sectional study from the Epidemiological Survey of Thyroid Diseases in Fujian Province, China. A total of 193 subjects with SCH (90 males and 103 females) without a history of treatment of thyroid disease, such as surgery, radiation, and thyroid hormone or antithyroid medication, were included in the present study. Indices of obesity, including body mass index (BMI), waist circumference (WC), and waist-height ratio (WHtR) were measured.

**Results:**

Our results showed that the secretory capacity of the thyroid gland (SPINA-GT) and Jostel's thyrotropin index (TSHI) were negatively correlated with BMI, WC, and WHtR, whereas the reciprocal of the thyrotroph thyroid hormone resistance index (TTSI-1) was positively correlated with BMI (all *p* < 0.05). After adjustment for age, sex, smoking, iodine status, and glucolipid metabolism, the associations between TSHI, TTSI (reciprocal transformation), and BMI still persisted (all *p* < 0.05).

**Conclusions:**

These results suggest that low levels of thyroid homeostasis indexes may be associated with overall obesity in SCH, rather than central adiposity.

## 1. Introduction

Subclinical hypothyroidism (SCH) is deﬁned as increased serum thyrotropin (TSH) levels with normal plasma free thyroxine (fT4) concentrations. The incidence of SCH varies from 4.6% to 16.7% in the population around the world, which is related to age and sex [1]. Previous studies have evaluated a possible association between SCH and obesity [2, 3]. SCH has been shown to have an increased prevalence in morbidly obese patients and is mostly corrected after weight loss (due to dieting or bariatric surgery); therefore, it has been speculated that SCH may be a consequence of abnormal fat accumulation and not a true hypothyroid state in morbidly obese subjects [4–7]. In addition, obesity complicated by SCH manifests higher insulin levels and low-grade inflammation in patients compared with obesity alone with normal TSH [8]. A growing body of evidence suggests that SCH has an important clinical impact on other metabolic abnormalities, including unfavorable lipoprotein profile [9, 10], atherosclerosis [11, 12], and endothelial dysfunction [13, 14]. These previous studies suggest that bidirectional effects exist between SCH and obesity.

The emergence of thyroid homeostatic indexes has improved our understanding of thyroid function and pituitary-thyroid feedback control [15–17], these include the calculated secretory capacity of the thyroid gland (SPINA-GT), Jostel's TSH index (TSHI), and thyrotroph thyroid hormone resistance index (TTSI), for assessment of thyrotropic pituitary function. Previous studies have suggested that these new qualitative and quantitative indexes have been translated into applications for clinical decision-making or research in thyroid disease [15, 18]. A continuous interaction between the thyroid hormone and regulatory mechanisms localized in the brain is important for human body weight control and maintenance of optimal energy balance [19]. Whether obesity has an effect on the pituitary-thyroid axis in SCH remains unknown. Identifying and understanding potential mechanisms linking SCH and obesity will better inform future treatment and prevention efforts for patients with SCH. Therefore, the current study examines whether thyroid homeostasis is related to obesity in a SCH population, independently of age, sex, smoking, iodine status, and glucolipid metabolism.

## 2. Materials and Methods

### 2.1. Participants

SCH participants consisted of a community-based cross-sectional research study from 2016 in the Fujian Province of China. All the participants enrolled in the national epidemiological survey (Tide) aimed to determine the prevalence of thyroid diseases and diabetes and the iodine nutrition status. A total of 2,651 individuals were randomly selected from the general population in urban (*n* = 1394) and rural (*n* = 1257) areas. All participants of the study were Han National residents, over 18 years of age, and living in Fujian for at least 5 years. The following were exclusion criteria: 1) patients that have received an iodine contrast agent examination or consumption of iodine-containing drugs within the past three months and 2) and pregnant or breastfeeding women. All subjects gave written informed consent in accordance with the Declaration of Helsinki. The protocol was approved by the Ethics Committee of Fujian Medical University Union Hospital (Grant no. 2015KY032). Subjects with high TSH levels (>4.2 mIU/L) and normal free T4 (FT4) levels (12.0–22.0 pmol/L) were diagnosed with SCH (*n* = 214). Patients with a history of thyroid disease treatment, such as surgery, radiation, and thyroid hormone or antithyroid medication were further excluded (*n* = 21). Finally, 193 subjects were included, including 90 males and 103 females.

### 2.2. Assessment of Demographic and Anthropometric Variables

All participants completed questionnaires to obtain disease history, smoking status, type of salt intake, demographic data, and medication use data. Smoking was defined as smoking at least one cigarette a day now or in the past, regardless of whether the subjects had quit smoking or not. The type of salt intake was defined as iodized or noniodized salt. Height and weight were measured in light clothing without shoes. Waist circumference (WC) was measured using a tape measure placed halfway between the lower border of the ribs and the iliac crest in a horizontal plane. Height, weight, and WC were measured twice and the averages were taken. Body mass index (BMI) was calculated by dividing the participant's weight (kg) by the square of height (m^2^). Waist-height ratio (WHtR) was calculated as WC (cm)/height (cm).

### 2.3. Clinical Examination and Laboratory Methods

Venous blood samples were collected between 8:00 a.m. and 10:00 a.m. after fasting overnight. Serum stored at −20°C was tested for thyroid hormones, including thyroid-stimulating hormone (TSH), free thyroxine (fT4), and free triiodothyronine (fT3) which were quantiﬁed using the immunochemiluminometric assay (ICMA) method on a Cobas 601 analyzer (Roche Diagnostic, Switzerland), using the standard kits from Roche Diagnostics GmbH (Germany). To screen the prevalence of thyroid disorders in the population, fT4 and fT3 were measured only among those with abnormal levels of TSH. The fT3, fT4, and TSH had a reference range of 3.1–6.8 pmol/L, 12–22 pmol/L, and 0.27–4.2 mIU/L, respectively. Midstream urine samples were collected in the morning and stored at −20°C. Urinary iodine concentration (UIC) was quantiﬁed using the ammonium persulfate method. Serum total cholesterol (TC), triglyceride (TG), high-density lipoprotein cholesterol (HDL-C), low-density lipoprotein cholesterol (LDL-C), and fasting plasma glucose levels were measured by an automated procedure (BS180; Mindray, Ltd., Shenzhen, China) immediately. Hemoglobin (Hb) A1c was measured by High-Performance Liquid Chromatography (HPLC) using an automated analyzer (VARIANT™ II TURBO, BioRad, Berkeley, USA) immediately.

### 2.4. Thyroid Homeostasis Assessment

The function of thyroid secretions was evaluated as SPINA-GT and calculated as SPINA-GT:

(1) $$\begin{array}{c} \hat{G}=\frac{\beta_{T}\;\left(\;D_{T}+\left[\text{TSH}\right]\;\right)\;\left(1+K_{41}\left[\text{TBG}\right]+K_{42}\left[\text{TBPA}\right]\;\right)\;{\left[\text{FT}\right]}_{4}}{\alpha_{T}\left[\text{TSH}\right]}, \end{array}$$

where *β*_*T*_ = 1.1 × 10^−6^/s, *D*_*T*_ = 2.75 mU/L, K_41_ = 2.0 × 10^10^ L/mol, TBG = 300 nmol/L, *K*_42_ = 2.0 × 10^8^ L/mol, TBPA = 4.5 *μ*mol/L, and *α*_T_ = 0.1/L. The function of the pituitary gland was evaluated by TSHI with an accurate estimate of the severity of pituitary dysfunction using the following formula [15]:

(2) $$\begin{array}{c} \text{TSHI}=\ln\left(\;\left[\text{TSH}\right]\;\right)+\beta \left[{\text{FT}}_{4}\right], \end{array}$$

where *β* = 0.1345 [16].

An additional index used to assess thyrotropic function and TSH feedback inhibition is TTSI, which was calculated by the following equation:

(3) $$\begin{array}{c} \text{TTSI}=\frac{100\left[\text{TSH}\right]\left[{\text{FT}}_{4}\right]}{l_{u}}, \end{array}$$

where *l*_*u*_ is the upper reference limit for fT4 [17].

### 2.5. Statistics

Statistical analysis was performed using SPSS, version 25.0 (SPSS Inc., IBM, Somers, NY). The Kolmogorov–Smirnov test was used to assess the normality of data distribution. The demographic characteristics and laboratorial parameters were summarized for men and women as the means ± SD for normally distributed continuous variables or as the medians and interquartile ranges (IQR) for nonnormally distributed continuous variables. Categorical variables were represented by percentage. Student's *t*-test was used to analyze the differences in means between males and females for variables with normal distribution. The Wilcoxon rank-sum test was used to compare the means for variables with nonnormal distribution. Chi-squared test or Fisher's exact test were performed to assess differences in categorical variables between the two groups. Because TTSI levels were distributed in a skewed manner, they were reciprocally transformed before statistical analysis. The reciprocal of TTSI was taken and the normality of TTSI^−1^ was assessed. It was found that it conforms to normal distribution. Crude relationships between thyroid homeostasis variables and obesity parameters were calculated using the Pearson correlation coefficient. Multiple linear regression analysis to evaluate the association between thyroid homeostasis variables and obesity parameters was adjusted for age, sex, smoking, type of salt intake, and UIC in Model 1 and additionally adjusted for TG, TC, HDL-C, fasting glucose, and HbA1c in Model 2. An analysis of residuals was performed for each model to assess the validity of assumptions of normality, homoscedasticity, and independence between observations (with the Durbin–Watson test). The tolerance indices were analyzed to check for possible multicollinearity. *p* < 0.05 was considered to be statistically significant.

## 3. Results

### 3.1. Descriptive Characteristics of Study Participates

The clinical characteristics of study participants, including 90 males and 103 females, are shown in Table 1. The mean age of males was 41.1 ± 1.6 years, and the mean age of females was 44.0 ± 1.7 years. Males had higher rates of current smoking and a larger waist circumference than females, whereas there was no difference in BMI and WHtR between males and females. Males showed significantly higher mean values for TG, fT4, and SPINA-GT, with lower HDL-C and TSH compared with females. There was no distinction in iodine status (iodized salt intake and UIC) between males and females.

### 3.2. Associations between Thyroid Homeostasis and Obesity

**Figure 1:**
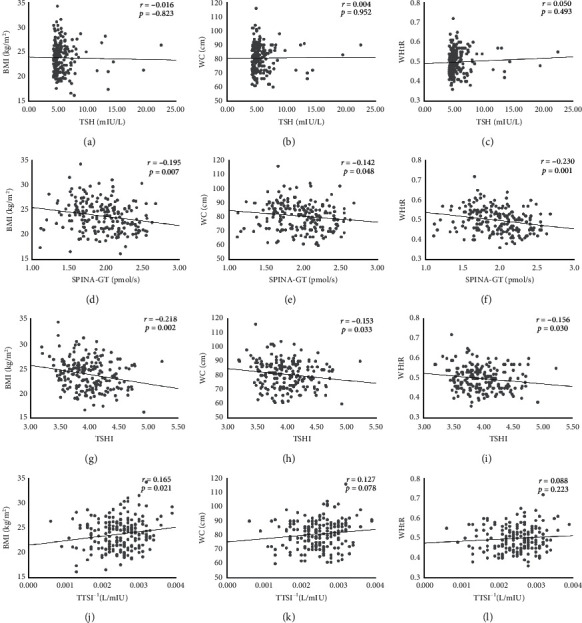
The relationship of thyroid homeostasis variables and obesity parameters. BMI: body mass index; WC: waist circumference; WHtR: waist-height ratio; SPINA-GT: secretory capacity of the thyroid gland; TSHI: Jostel's TSH index; TTSI: thyrotroph thyroid hormone resistance index.

The mean of SPINA-GT was 1.95 ± 0.02 pmol/s, and it was significantly decreased with BMI (*r* = −0.195, *p*=0.007), WC (*r* = -0.142, *p*=0.048), and WHtR (*r* = −0.230, *p*=0.001). TSHI (3.98 ± 0.03) was negatively correlated with BMI (*r* = -0.218, *p*=0.002), WC (*r* = −0.153, *p*=0.033), and WHtR (*r* = −0.156, *p*=0.030). TTSI^−1^ was positively correlated with BMI (*r* = 0.165, *p*=0.021). However, the correlation between TSH and BMI, WC, and WHtR was not statistically significant (*p*=0.431, *p*=0.687, *p*=0.090, respectively). In addition, the correlation between TTSI^−1^ and WC and WHtR was not statistically significant (*p*=0.078, *p*=0.223, respectively) (Figure 1).

### 3.3. Independent Associations between Thyroid Homeostasis and Obesity

In model 1 adjusted for age, sex, smoking, type of salt intake, and UIC, multiple linear regression analysis revealed that TSHI was negatively associated with BMI (*β* = −0.162; p = 0.015), and TTSI^−1^ was positively associated with BMI (*β* = 0.144; *p*=0.027). Upon forcing TG, TC, HDL-C, fasting glucose, and HbA1c into the model (model 2), the correlation between TSHI and TTSI^−1^ with BMI remained significant (*β* = −0.139, *p*=0.024; *β* = 0.143, *p*=0.018, respectively). However, SPINA-GT was not correlated with obesity parameters under either model 1 or model 2 (Table 2).

## 4. Discussion

The main finding of the present study was that a significant correlation exists between thyroid homeostasis and obesity in study participants with SCH. Moreover, this association was independent from the effects of potential confounders, such as age, sex, smoking, iodine status, and glucolipid metabolism. However, most associations were attenuated after adjustment, suggesting a potential mediatory role for thyroid homeostasis. To the best of our knowledge, a few studies have attempted to evaluate participants with SCH using a large, community-based population to identify an independent association between thyroid homeostasis and obesity indexes.

Thyroid function has been extensively investigated in obese subjects with the purpose of relating an increase in body weight to an underlying thyroid disturbance [19, 20]. The measurement of TSH, although an indirect indicator of thyroid homeostasis, has become the standard of contemporary thyroid function testing [21, 22]. A large and national survey observed a signiﬁcant positive association between serum TSH and FT3 (to a lesser degree) in a euthyroid population, with both BMI and waist circumference, whereas no association with FT4 could be demonstrated [23]. A meta-analysis confirmed that a high-normal serum TSH was associated with a high BMI [24]. It has been observed that TSH normalization after weightlessness was caused by a low-calorie diet or weight loss surgery [4–7], suggesting that TSH elevation in obese patients is an adaptive response by the hypothalamus-pituitary-thyroid axis to weight gain. The alterations in body weight associated with overt hypothyroidism may reflect both the accumulation of body fat, due to decreased resting energy expenditure (REE) and reduced physical activity, and the increased water content of the body, consequent to a reduced capacity to excrete free water [19]. However, the relationship between serum levels of TSH and indices of obesity in SCH remains unknown. BMI has not been found to be higher in elderly women with SCH compared with euthyroid controls [25]. In a previous study, patients with SCH showed a significantly higher BMI than controls [26]. In our study, we observed no correlation between TSH and obesity index (BMI, WC, and WHtR) in a SCH population.

Previous studies have shown that TSH levels defined for optimum health may not apply equivalently during treatment with L-thyroxine (L-T4), suggesting that universal reference ranges for TSH and peripheral thyroid hormones may not be appropriate [27]. Frequent divergencies between composite multivariate reference limits and a combination of separate univariate reference intervals challenge the validity of the conjoined roles of TSH [28]. Thyroid homeostasis parameters (SPINA-GT, TSHI, and TTSI) based on mathematical modeling of pituitary-thyroid feedback control have provided functional insights beyond the single variable reference range [15, 27, 29]. Our research further investigated the correlation between thyroid homeostasis parameters (SPINA-GT, TSHI, and TTSI) and BMI, WC, and WHtR in SCH patients and provided more clinical evidence between SCH and obesity.

The most common endogenous cause of SCH is chronic autoimmune thyroiditis associated with antithyroid peroxidase antibodies [1]. Although there are little data on thyroid structural changes in obese patients, the gland volume of obese patients is larger than nonobese subjects assessed by ultrasound (US) [30]. SPINA-GT provides better functional measurements than thyroid volume because scarring and thyroid tissue damage are common in thyroid autoimmune diseases [18]. SPINA-GT, as a calculated parameter, denotes the maximum amount of T4 that the thyroid can produce in a given time unit under stimulated conditions. The reliability of SPINA-derived parameters is higher than measured hormone concentrations [15]. Conversely, two indices of standardized pituitary TSH response were introduced for diagnosing pituitary insufficiency (TSHI) and thyroid hormone resistance (TTSI). TSHI defines the maximum possible TSH response in the fT4-uninhibited state at a theoretical fT4 value of 0, whereas the TTSI standardizes the TSH response in relation to the upper reference range of FT4. Both indices define the pituitary thyrotroph function more accurately compared with TSH measurement and proved to be useful for clinical disease classification [16, 17]. A previous observational study showed a higher TSHI and a lower SPINA-GT in SCH obese patients compared to obese patients with normal TSH [8]. Another study revealed that thyroid autoimmunity is not a major cause of SCH, suggesting that the high rate of SCH could be related to morbid obesity (SCH patient BMI > 40 kg/m^2^) [31]. The underlying mechanisms leading to the association between SCH and obesity remain unknown. In our study, obesity indices (BMI, WC, and WHtR) and thyroid homeostasis were assessed continuously throughout the study to avoid the use of a cut-off. We found that SPINA-GT and TSHI were negatively correlated with BMI, WC, and WHtR, whereas TTSI^−1^ was positively correlated with BMI, which suggested that obese SCH patients had a worse thyrotropic pituitary function and thyroid gland secretory capacity. After adjustment for age, sex, smoking, iodine status, and glucolipid metabolism, select associations persisted.

As BMI cannot differentiate between lean and fat mass, the use of WC and WHtR in our study may be a more discriminating index of central adiposity. Remarkably, after adjustment for these additional confounders (both Model 1 and Model 2), all associations between SPINA-GT, TSHI, TTSI, and WC, WHtR disappeared. These discrepancies could be partially explained by epidemiological aspects. First, central adiposity is regarded as an estimate of cardiometabolic risk factors in a variety of populations [32–35]. Second, it was well known that age, sex, smoking, and glucolipid metabolism are independent risk factors of cardiovascular disease. Therefore, these risk factors may weaken the association between thyroid homeostasis and central adiposity.

The present study had several limitations. First, the cross-sectional design of the study does not clarify whether relatively low levels of thyroid homeostasis indexes are the consequence or the cause of obesity in participants with SCH. This is a critical issue because, in the latter case, small variations in thyroid homeostasis indexes might have negative consequences on body weight and eventually on metabolic and cardiovascular outcomes. Second, due to the diurnal variation of thyroid hormone levels, the lack of repeated assessment of thyroid hormone levels may lead to some errors in measurement. However, all blood samples in the present study were taken continuously in the morning after a night of fasting, to minimize such errors. Third, clinical and genetic evidence supports the view that obesity does not represent a persistent entity and that morbidly obese (BMI > 40 kg/m^2^) patients may suffer from different diseases compared with mildly overweight patients [31]. In our study, participants that were morbidly obese were not included (maximum BMI = 34.3 kg/m^2^), suggesting that our results may not apply to all obese populations. A further limitation may be that the regression coefficients of BMI to TSHI and TTSI^−1^ are relatively small. However, although the correlation was weak, BMI was independently associated with TSHI and TTSI.

## 5. Conclusions

In conclusion, data gathered from a community-based and representative cohort demonstrated that SPINA-GT and TSHI were negatively associated with BMI in participants with SCH independently of age, sex, smoking, iodine status, and glucolipid metabolism. Further research is required to investigate whether or not aggressive management, including repeated evaluation for thyroid homeostasis or thyroid hormone replacement therapy for persons with SCH, can improve adiposity status and metabolism.

## Figures and Tables

**Table 1:** Clinical characteristics of study participants by sex.

Characteristics	All (*n* = 193)	Male (*n* = 90)	Female (*n* = 103)	^*∗*^ *p* value
Age (years)	42.6 ± 1.2	41.1 ± 1.6	44.0 ± 1.7	0.197
Smoking (*n*, %)	29 (15.0%)	28 (31.1%)	1 (1.0%)	**<0.001**

*Iodine status*				
Iodized salt intake (*n*, %)	173 (89.6%)	82 (91.1%)	91 (88.3%)	0.530
UIC (*μ*g/L)	152 (100–234)	163 (111–255)	142 (89–228)	0.085

*Obesity indexes*				
BMI (kg/m^2^)	23.8 ± 3.1	24.1 ± 3.1	23.5 ± 3.1	0.231
WC (cm)	80.8 ± 9.7	83.9 ± 9.6	78.1 ± 9.0	**<0.001**
WHtR	0.499 ± 0.061	0.499 ± 0.059	0.498 ± 0.063	0.917

*Glucolipid metabolism*				
TG (mmol/L)	1.07 (0.80–1.61)	1.17 (0.82–2.10)	1.01 (0.74–1.36)	**0.026**
TC (mmol/L)	5.01 (4.29–5.79)	4.95 (4.16–5.75)	5.13 (4.36–5.86)	0.326
LDL-C (mmol/L)	2.48 (2.04–2.98)	2.55 (2.05–2.94)	2.44 (2.02–3.01)	0.673
HDL-C (mmol/L)	1.30 (1.11–1.58)	1.19 (1.05–1.45)	1.37 (1.17–1.69)	**0.001**
Glucose (mmol/L)	5.20 (4.84–5.58)	5.19 (4.74–5.54)	5.22(4.91–5.59)	0.413
HbA1c (%)	5.7 (5.4–6.1)	5.7 (5.3–6.0)	5.8 (5.4–6.3)	0.476

*Thyroid related hormones*				
TSH (mIU/L)	5.01 (4.60–6.11)	4.84 (4.55–5.58)	5.30 (4.63–6.28)	**0.044**
fT4 (pmol/L)	16.9 ± 0.2	17.5 ± 0.3	16.4 ± 0.2	**0.002**

*Thyroid homeostasis*				
SPINA-GT (pmol/s)	1.95 ± 0.02	2.04 ± 0.03	1.88 ± 0.03	**0.001**
TSHI	3.98 ± 0.03	4.02 ± 0.04	3.94 ± 0.03	0.103
TTSI (mIU/L)	394 (351–465)	397 (353–462)	389 (349–469)	0.979

^*∗*^ *p* values of males and females. Data are shown as mean (SD), median (interquartile range), or percentage. BMI: body mass index; WC: waist circumference; WHtR: waist-height ratio; SPINA-GT: secretory capacity of the thyroid gland; TSHI: Jostel's TSH index; TTSI: thyrotroph thyroid hormone resistance index; UIC: urinary iodine concentration; TG: triglyceride; TC: total cholesterol; LDL-C: low-density lipoprotein cholesterol; HDL-C: high-density lipoprotein cholesterol; HbA1c: hemoglobin A1c.

**Table 2:** Multiple linear regression analysis to determine the association between thyroid homeostasis variables and obesity parameters.

	BMI		WC		WHtR	
	*β*	*p* value	*β*	*p* value	*β*	*p* value
*SPINA-GT*						
Model 1	−0.088	0.207	−0.070	0.271	−0.048	0.426
Model 2	−0.019	0.772	−0.010	0.872	0.001	0.987

*TSHI*						
Model 1	−0.162	**0.015**	−0.108	0.077	−0.056	0.338
Model 2	−0.139	**0.024**	−0.082	0.144	−0.034	0.537

*TTSI* ^−1^						
Model 1	0.144	**0.027**	0.099	0.098	0.054	0.340
Model 2	0.143	**0.018**	0.093	0.091	0.048	0.374

Model 1: adjusted for age, sex, smoking, type of salt intake, and UIC. Model 2: adjusted for age, sex, smoking, type of salt intake, UIC, TG, TC, HDL-C, fasting glucose, and HbA1c. BMI: body mass index; WC: waist circumference; WHtR: waist-height ratio; SPINA-GT: secretory capacity of the thyroid gland; TSHI: Jostel's TSH index; TTSI: thyrotroph thyroid hormone resistance index; UIC: urinary iodine concentration; TG: triglyceride; TC: total cholesterol; HDL-C: high-density lipoprotein cholesterol; HbA1c: hemoglobin A1.

## Data Availability

The data used to support the findings of this study are available from the corresponding author upon request.
